# Primary appendiceal Burkitt's lymphoma presenting as acute appendicitis: An extremely rare case report and review of the literatture

**DOI:** 10.1016/j.amsu.2020.11.083

**Published:** 2020-12-14

**Authors:** Abdelilah El Bakouri, Ahmed Ballati, Mounir Bouali, Khalid Elhattabi, Fatimazahra Bensardi, Abdelaziz Fadil

**Affiliations:** a Department of General Surgery, University Hospital Centre Ibn Rochd, Casablanca, Morocco; b Faculty of Medecine and Pharmacy, Hassan II University, Casablanca, Morocco

**Keywords:** Burkitt's lymphoma, Acute appenditis, Chemotherapy, Surgery

## Abstract

Primary lymphomas of appendix are extremely rare tumors. The incidence is 0.015% out of all gastrointestinal lymphomas; furthermore, limited data is available in literature. The appendiceal neoplasms are most commonly presented as acute appendicitis followed intestinal obstruction, intussusception or perforation. We present a case of a 22 year-old male patient who presented with acute appenditis and underwent emergency laparotomy. On abdominal exploration, swollen and enlarged appendix measured 3cm was present for which appendectomy were performed. The histopathological examination of appendectomy specimen revealed a Burkitt's Lymphoma. The patient received R-COPADEM protocol of chemotherapy. Primary gastrointestinal lymphoma is a extremely rare neoplasm without guidelines for therapy.

## Highlights

• Burkitt lymphoma is an aggressive non-hodgkin b-cell lymphoma.
• The occurrence of primary Burkitt's lymphoma of appendix is extremely rare.
• The appendiceal neoplasms are most commonly presented as acute appendicitis.
• The diagnosis of kimura disease is confirmed by surgical biopsy.
• The diagnostic of Burkitt's lymphoma was only made after histopathological examination, so should be done.
• The surgery and chemotheray constitue a best traitement for burkitt's lymphoma of appendix presenting as acute appendicitis.

## 1 Introduction

Primary appendix neoplasms are estimated to be less than 1.0% of appendectomy specimens. Burkitt's lymphoma is a mature B-cell non-Hodgkin lymphoma. Only 1–4% of gastrointestinal tract tumors are classified as primary Non Hodgkin Lymphomas [1,2]. The largest reported study on appendiceal lymphomas involved 116 patients out of which 25.9% were Burkitt lymphomas. Due to the difficulty of preoperative diagnosis, it is often essential to conduct histopathological examinations of all appendectomy [3]. This case is reported in line with the SCARE criteria [4].

### 1.1 Case description

A 22-year-old man presented with a 48-h history of abdominal pain located in the lower right quadrant. His physical examination revealed tenderness in the right lumbar and right iliac fossa, consistent with signs of acute appendicitis. His body temperature was 39.5 °C. Laboratory examination revealed white cell count of 15,500/mm3. An abdominal ultrasonography revealed a mass in the right lower abdomen.

**Fig. 1:**
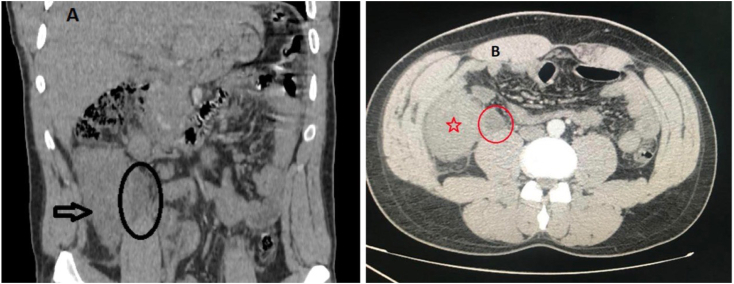
A. CT scan of the abdomen (coronal plane) B. CT scan of abdomen (axial plane) reveal an enlarged appendix (encircled black circle and red circle) and fluid. (For interpretation of the references to colour in this figure legend, the reader is referred to the Web version of this article.)

**Fig. 2:**
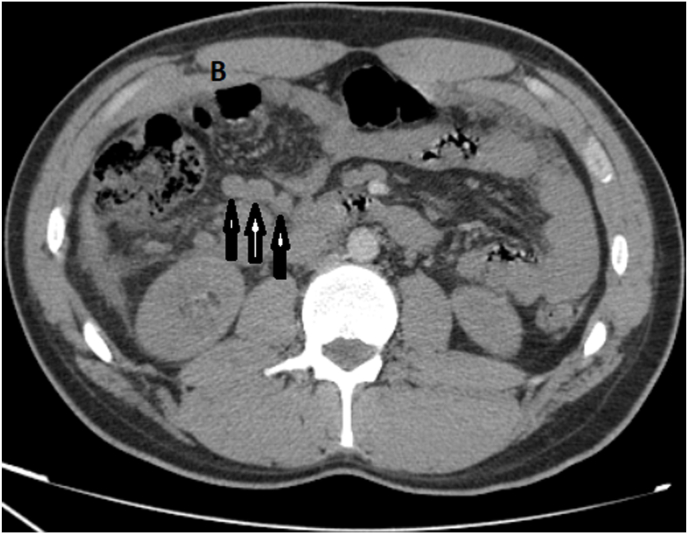
CT scan of abdomen (axial plane) reveal mesenteric and para-aortic lymphadenopathy (three vertical arrows).

**Fig. 3:**
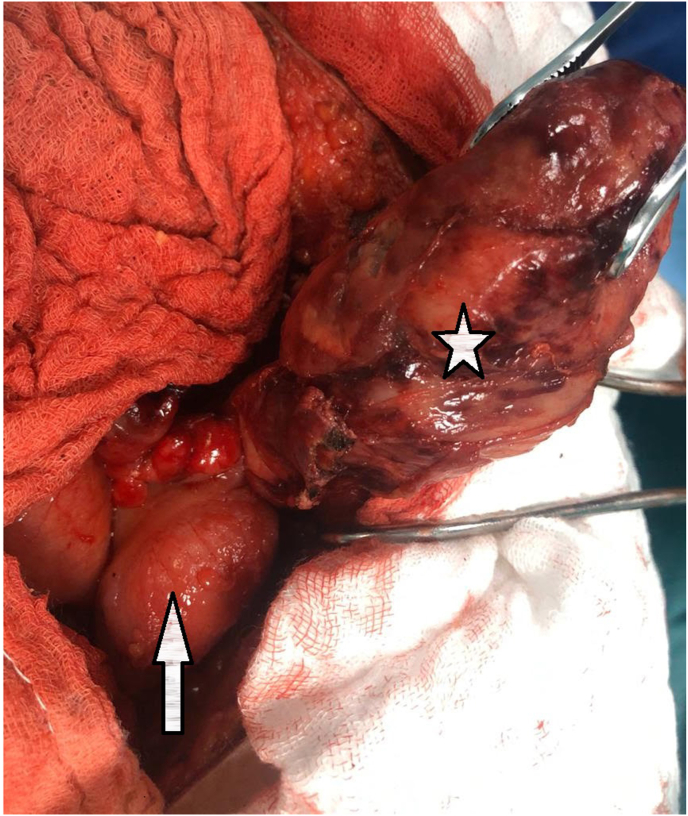
Intra operative picture of appendiceal tumor measured (white star) 8cm*4cm and normal caecum (whita arrow).

**Fig. 4:**
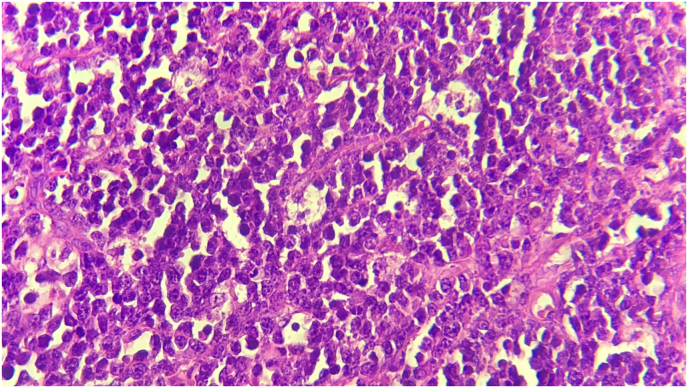
Numerous tingible body macrophages imparting the classic starry sky appearance were seen.

While abdominal CT/scan was perfomed and showed a fluid collection in the right iliac fossa measuring 6.5cm*6cm, a swollen appendix is identified reaching a diameter of 13 mm (Fig. 1), mesenteric and para-aortic lymphadenopathy (Fig. 2). A Midline incision revealed an appendicular mass measured 8cm*4cm (Fig. 3), so an appendectomy was performed. The postoperative period was regular. The discharge was on the 4 th postoperative day. The histopathological exam of the appendix was found diffuse and transmural lymphoid proliferation. Numerous tingible body macrophages imparting the classic starry sky appearance were seen (Fig. 4). Immunoperoxidase stains were positive for CD10, CD20, Bcl-6, marker of proloferation KI-67 were expressed in 100% of lymphoma cells and Bcl-2 were not expressed. We have concluded of diagnostic of Burkitt's lymphoma of the appendix. Further extensive investigation including staging PET/CT did not demonstrated metabolically active lesions. The patient received R-COPADEM protocol of chemotherapy. Follow up CT scans of chest and abdomen over 7 months shown no evidence locoregional recurrence, lymphadenopathy or metastasis after surgery and chemotherapy.

## 2 Discussion

The lymphomas can be divided in Hodgkin and non-Hodgkin and the Burkitt's lymphoma is an aggressive non-Hodgkin B-cell lymphoma [2]. The gastrointestinal tract lymphoma represents 4–20% of non-Hodgkin's lymphoma and 30–45% of extranodal cases. The stomach is most commonly affected, followed by the small bowel, pharynx and colon. The median age at diagnosis for gastrointestinal tract Non Hodgkin Lymphoma is 55 years. The disease is empirically more present in men. Acute appendicitis is typical for most appendicular tumors [5]. Other uncommon symptoms include gastrointestinal bleeding, and intussusception. Appendiceal tumor is generally an intraoperative diagnosis. When suspected preoperatively, chest, abdomen and pelvis scan is a convenient method that can reveal a diffuse enlargement of the appendix with thickening of the wall and periappendiceal tissue, which potentially suggests lymphomatous infiltration or periappendiceal inflammation [6]. It may change surgical strategy when detect neoplasms with preoperative imaging [7]. On CT, the abdominal lymphadenopathy and diameter of the lymphomatous appendix usually measures 3 cm or larger, which is way larger than the expected size for non-tumoral appendicitis, hence can lead to a presumptive diagnosis of appendiceal neoplasm [8]. In our case, the CT scan revealed an abdominal lymphadenopathy, however the appendix diameter was not thick enough to indicate an appendiceal neoplasm. The diagnosis of Burkitt's Lymphoma was only made after histopathological examination, so should be done. The role of PET/CT in lymphoma is very important for staging and metabolic response to therapy. PET/CT has high sensitivity, in identifying lymph node and extranodal that involves lymphoma [9]. In our case,PET/CT, did not identify metabolic activity. Given the scarcity of the studies on Burkitt's Lymphoma of appendix as well as the low incidence rate, it is hard to find guidelines for the best approach. The largest reported case series involved 116 patients with primary appendiceal lymphomas, this study did not reveal a significant difference in the survival rate between appendectomy and hemicolectomy [3]. In our case, appendectomy was performed, and post-operative R-COPADEM protocol was applied.

## 3 Conclusion

Primary lymphoma of the appendix is an extremely rare pathology. there are unclear guidelines for treatment. Acute appendicitis is a frequent presentation of primary appendiceal lymphoma. All appendectomies must be undergo the histopathological examination. The surgery and chemotherapy constitute a best treatment for Burkitt's lymphoma of appendix presenting as acute appendicitis.

## Ethical approval

I declare on my honor that the ethical approval has been exempted by my establishment.

## Author contribution

Ahmed Ballati: Corresponding author writing the paper, Abdelilah Elbakouri: writing the paper, Mounir Bouali: study concept, Khalid Elhattabi: correction of the paper, Fatimazahra Bensardi: correction of the paper, Abdelaziz Fadil: correction of the paper.

## Consent

Written informed consent for publication of their clinical details and/or clinical images was obtained from the patient’s parents.

## Registration of Research Studies

Researchregistry5198.

## Guarantor

DOCTEUR AHMED BALLATI

## Funding

None.

### Provenance and peer review

Not commissioned, externally peer reviewed.

## Declaration of competing interest

The authors declare having no conflicts of interest for this article
